# Novel allyl-hydrazones including 2,4-dinitrophenyl and 1,2,3-triazole moieties as optical sensor for ammonia and chromium ions in water

**DOI:** 10.1186/s13065-022-00820-2

**Published:** 2022-04-07

**Authors:** Hanan A. Mohamed, Bakr F. Abdel-Wahab, Mahmoud N. M. Yousif, Reda M. Abdelhameed

**Affiliations:** 1 Applied Organic Chemistry Department, Chemical Industries Research Institute, National Research Centre, Scopus Affiliation ID 60014618, 33 EL Buhouth St., Dokki, Giza, 12622 Egypt; 2 Photochemistry Department, Chemical Industries Research Institute, National Research Centre, Scopus Affiliation ID 60014618, 33 EL Buhouth St., Dokki, Giza, 12622 Egypt

**Keywords:** 1,2,3-triazoles, Allyl hydrazones, Aldol condensation, (2,4-dinitrophenyl)hydrazine, Heavy metal sensor, Ammonia sensor

## Abstract

It is critical to take safety action if carcinogenic heavy metals and ammonia can be detected quickly, cheaply, and selectively in an environmental sample. As a result, compound 4a [4-(1-(2-(2,4-Dinitrophenyl)hydrazineylidene)-3-(naphthalen-2-yl)allyl)-5-methyl-1-phenyl-1* H*-1,2,3-triazole] and compound 4b [4-(1-(2-(2,4-Dinitrophenyl)hydrazineylidene)-3-(naphthalen-2-yl)allyl)-1-(4-fluorophenyl)-5-methyl-1* H*-1,2,3-triazole] were prepared. The aldol condensation process of 4-acetyl-1,2,3-triazoles 1a,b (Ar = C_6_H_4_; 4-FC_6_H_4_) with 2-naphthaldehyde yields 1-acetyl-1,2,3-triazoles 1a,b (Ar = C_6_H_4_; 4-FC_6_H_4_) (5-methyl-1-aryl-1 H-1,2,3-triazol-4-yl) -3-(naphthalen-2-yl)prop-2-en-1-ones 3a,b with a yield of around 95%. The target compounds 4a,b are obtained in around 88% yield by condensation of 3a,b with (2,4-dinitrophenyl)hydrazine in a refluxing acidic medium. Compounds 4a,b exhibited possible colorimetric detection for chromium ion in the range of 0–14 ppm and ammonia in the range of 0–20 ppm. As a result, this research suggests that strong electron-withdraw groups in related probes can improve anion detection ability, while the conjugation effect should also be considered while building structures.

**Supplementary Information:**

The online version contains supplementary material available at 10.1186/s13065-022-00820-2.

## Introduction

Hydrazone derivatives have gained a lot of attention due to their commercial use as dyestuffs [1], antibacterial agents [2, 3], antiviral agents [4, 5], and anticancer agents [6, 7]. Furthermore, hydrazone has a wide range of applications in the construction of sensor materials. It was used to detect fluoride ions, cyanide ions, heavy metals, and poisonous fumes, among other things. In the selective and sensitive detection of fluoride ions, an indole hydrazone tagged moiety, 2-((5-bromo-1 H-indol-2-yl) methylene) hydrazono) methyl)-4, 6-diiodophenol, was utilized. The sensing method involves an increase in a fluorescence band at 430 nm and the simultaneous disappearance of the emission band at 555 nm due to a deprotonation process triggered by the development of a hydrogen-bonding complex [8]. The detection limit for fluoride ions in the organic and aqua organic medium is 0.45 and 0.41 ppm, respectively, for isatin hydrazones with hydroxy and amine groups acting as binding sites for sensitive and selective sensing of fluoride ions in 100% acetonitrile and 20% aqua acetonitrile media [9]. Acyl hydrazone such as *N*′-[(1Z)-1-(4-fluorophenyl)ethylidene]benzohydrazide was synthesized and used as a sensor for fluoride ions [10]. Hydrazone Schiff bases were made from 1,8-naphthalimide hydrazide and substituted furan and thiophene rings, and the generated compounds were used in fluoride ion detection with a quick response and fluorescence quenching [11].

The color of hydrazones with CN, CF_3_, and NO_2_ derivatives changed from colorless to varied shades of blue when they reacted with cyanide ions, indicating that they are sensitive to cyanide ions. The cyanide ion detection limit for commonly used hydrazones is 0.0477 µM [12]. For quick, selective, and sensitive detection of cyanide ions in an aqueous medium, (E)-2-(2-(thiophen-2-ylmethylene)hydrazinyl)-4,5-dihydro-1 H-imidazol-3-ium bromide was utilized. The detection limit was discovered to be 0.89 µM [13]. The benzamide hydrazone sensor may detect cyanide ions with a very low detection limit [14].

The fluorescent “turn-on” detection of Al^3+^ in semi-aqueous solutions was achieved using hydrazone, which was made from pyrazine-2-carbohydrazide and 1-phenyl-3-methyl-4-benzoyl-5-pyrazolone. For Al^3+^, the sensor has detection limits of 0.18 µM [15]. The colour of ethyl(E)-4-(2-(3-methyl)ethyl(E)-4-(2-(3-methyl)ethyl(E)-4- With Co^2+^, Zn^2+^, and Cu^2+^, the colour of -4-((E)-phenyldiazenyl)-1 H-pyrazol-5-yl)hydrazono)-5-oxo-2-phenyl-4.5-dihydro-1 H-pyrrole-3-carboxylate was changed from red to violet, olive, and green, respectively. The detection limit for Co^2+^, Zn^2+^, and Cu^2+^ was found to be between 0.59 and 0.98 µM [16]. Research on multidrug resistance plays a vital role in designing chemicals for drug uses, at present the new sulfonamide can be chelated with the metal for this purpose [17]. The fluorescent “On–Off” sensor for Hg^2+^ ion was phenothiazine-thiophene hydrazone, such as 10-ethyl-10 H-phenothiazine-3,7-diyl)bis(methanylylidene))bis(thiophene-2-carbohydrazide. The value of the limit of detection (LOD) was discovered to be 0.44 108 M [18]. By monitoring variations in absorption and fluorescence spectrum patterns, a quinoline-based hydrazone such as bis((quinolin-8-yl)methylene)carbonohydrazide was utilised as a dual probe for selective identification of Co^2+^ and Zn^2+^. The detection limits for Co^2+^ are 0.21 M and for Zn^2+^ are 0.66 M [19]. Heavy metal ion sensing was shown using ferrocenyl hydrazone and rhodamine [20].

Sensing of nitro-explosives using hydrazones such as 1,1,2,2-tetrakis(4-formyl-(1,1′-biphenyl))ethane and 1,3,5-benzenetricarboxylic acid trihydrazide revealed a new sensing platform for nitro-explosives with high sensitivity (Ksv 106 M) and selectivity up to 99% [21]. The 4-pyridinecarboxylic acid hydrazone of (+/−) gossypol was obtained by reacting (+/−) gossypol isolated from cotton seeds with 4-pyridinecarbo hydrazide. The chemical was tested as a fluorescent probe to detect 2,4,6-trinitrophenol (TNP), 2,4-dinitrophenol (DNP), and 4-nitrophenol (NP) in solution. The molecule has the highest sensitivity (Ksv = 1.1 × 10^5^ M) and selectivity (LOD = 0.59) for TNP [22]. For acetate ion detection, hydrazones with an N–H group, such as (E)-1-(ferrocenyl)-2-(2,4-dinitrophenyl)hydrazine, were utilized. The hydrogen interaction between the chemical and the acetate ion is the sensing mechanism, with a detection limit of 0.84 ± 0.03 µM [23]. Tris(keto-hydrazone with secondary amines and alkoxy groups was utilized to detect harmful hydrogen sulphide (H_2_S) gas with a detection limit of 25 ppb in toxic gas sensing [24]. Hydrazone was chelated with Zn^2+^ using a nitro group donor set, resulting in particular fluorescence improvements for a hydrogen sulfide reversible on–off sensor with a detection limit of 32.6 nM [25].

Ammonia is one of the anions having a trigonal chemical structure that allows it to create strong hydrogen bonds with hydrogen-bond donors. Designing receptors that can recognize and sense the ammonia ion at very low levels is becoming increasingly crucial in this regard. The recognition of the ammonia ion, in the example, might be easily followed by a visual color shift, allowing for “naked-eyes” detection without the use of spectroscopic gear. As a result, our goal is to build promising hydrazones for selective ammonia ion sensing.

## Materials

The metal salts used in this investigation were of an analytical grade. Potassium chromate (K_2_CrO_4,_ 99% Aldrich), Zinc chloride anhydrous (ZnCl_2,_ 99.9% PURE), aluminum chloride (AlCl_3,_ 99.9% Merck), mercuric chloride (HgCl_2,_ 99.9% VEB BERLIN-CHEME), nickel chloride hexahydrate (NiCl_2_·6H_2_O, 99% PURE), Manganese chloride hydrate (MnCl_2_·4H_2_O, 99% BDH), and tin chloride dihydrate (SnCl_2_·H_2_O, 99% Laboratory Rusayan). The solvent used with high purity, *N,N*-Dimethylformamide (DMF, 99.8% Sigma-Aldrich), Ammonium hydroxide solution (NH_4_OH, 30% Sigma-Aldrich).

### Synthesis of 1-(5-Methyl-1aryl-1*H*-1,2,3-triazol-4-yl)-3-(naphthalen-2-yl)prop-2-en-1-ones 3a,b

Compounds **1a** or **1b** (5 mmol) were added to an ethanolic sodium hydroxide [sodium hydroxide (0.4 g, 10 mmol) in water (10 mL) and ethanol (30 mL)] was stirred at room temperature for 30 min. Then add 2-naphthaldehyde (0.78 g, 5 mmol) and the stirring was continued for an additional 3.5 h. Pour the resulting solution into ice water and complete the stirring for 30 min. Filter the solid product and wash with water and dry then crystallize from ethanol.

### Synthesis of 1-(5-Methyl-1-phenyl-1*H*-1,2,3-triazol-4-yl)-3-(naphthalen-2-yl)prop-2-en-1-one 3a

Compound **3a** was obtained as colorless solid (95%); mp 180–182 °C. ^1^H NMR (500 MHz, DMSO): δ *=* 2.65 (s, 3 H, CH_3_), 7.46 (d, 1H, CH, J = 8.6 Hz), 7.48, 7.49 (2s, 2H, Ar-H), 7.53 (d, 1H, CH, J = 8.6 Hz), 7.55–8.20 (m, 12H, Ar-H); ^13^ C NMR (125.7 MHz, DMSO): 10.45, 123.34, 124.29, 125.38, 126.21, 126.74, 127.39, 127.89, 128.76, 129.75, 130.10, 130.82, 132.63, 133.47, 134.52, 135.53,138.60, 143.76, 144.15, 184.35.

### Synthesis of 1-(1-(4-Fluorophenyl)-5-methyl-1*H*-1,2,3-triazol-4-yl)-3-(naphthalen-2-yl)prop-2-en-1-one 3b

Compound **3b** was obtained as colorless solid (93%); mp 160–161 °C. ^1^H NMR (500 MHz, DMSO): δ *=* 2.66 (s, 3H, CH_3_), 7.27 (d, 1H, CH, J = 9.2 Hz), 7.48, 7.51 (2d, 2H, J = 8.5 Hz Ar-H) 7.86 (d, 1H, CH, J = 9.2 Hz), 7.87–8.18 (m, 9H, Ar-H); ^13^C NMR (125.7 MHz, DMSO): 10.36, 116.80 (d, *J*_C−F_ = 23.85 Hz), 116.99, 123.19, 124.26, 126.75, 127.40, 127.47, 127.89 (d, *J*_C−F_ = 52.46 Hz), 128.76, 130.87, 131.60, 132.57, 133.47, 134.58, 143.94, 144.15 (d, *J*_C−F_ = 26.22 Hz), 162.31, 164.30 (d, *J*_C−F_ = 48.60 Hz), 184.31.

### Synthesis of 4-(1-(2-(2,4-Dinitrophenyl)hydrazineylidene)-3-(naphthalen-2-yl)allyl)-5-methyl-1-aryl-1*H* -1,2,3-triazoles 4a,b

To a solution of appropriate chalcones **3a** or **3b** (3 mmol) in ethanol (25 mL) and Conc. HCl (0.5 mL), 2,4-dinitrophenylhydrazine (3 mmol) was added. The reaction mixture was refluxed for 3 h. The formed solid was filtered and washed with ethanol and crystallized from DMF.

### Synthesis of 4-(1-(2-(2,4-Dinitrophenyl)hydrazineylidene)-3-(naphthalen-2-yl)allyl)-5-methyl-1-phenyl-1*H*-1,2,3-triazole 4a

Compound **4a** was obtained as orange solid (88%); mp 225–226 °C. ^1^H NMR (500 MHz, DMSO): δ *=* 2.64 (s, 3H, CH_3_), 7.25 (s, 1H, ArH), 7.52 (d, 1H, J = 7.65 Hz, CH), 7.63 (d, 1H, CH, J = 7.65 Hz), 7.64–8.01 (m, 13H, Ar-H), 9.17 (s, 1H, ArH), 11.70 and 12.56 (s, 1H, NH, D_2_O exchangeable); ^13^C NMR (125.7 MHz, DMSO): 10.80, 116.92, 117.35, 117.47, 123.44, 124.23, 126.63, 127.26, 127.74, 128.22, 128.44, 128.51, 128.71, 128.76, 128.94, 129.27, 129.75, 132.42, 133.76, 135.75, 138.09, 138.38, 144.42, 145.31, 162.19, 164.16.

### Synthesis of 4-(1-(2-(2,4-Dinitrophenyl)hydrazineylidene)-3-(naphthalen-2-yl)allyl)-1-(4-fluorophenyl)-5-methyl-1*H*-1,2,3-triazole 4b

Compound **4b** was obtained as orange solid (87%); mp 238–240 °C. ^1^H NMR (500 MHz, DMSO): δ *=* 2.53 (s, 3H, CH_3_), 7.48 (d, 1H, J = 7.65 Hz, CH), 7.52 (s, 1H, ArH), 7.83 (d, 1H, CH, J = 7.65 Hz), 7.93–8.16 (m, 12H, Ar-H), 8.84 (s, 1H, ArH), 11.78 and 12.57 (s, 1H, NH, D_2_O exchangeable); ^13^C NMR (125.7 MHz, DMSO): 10.80, 116.81, 116.92, 117.13, 117.29, 117.47 (d, *J*_C−F_ = 22.65 Hz), 124.23, 126.63, 127.26, 127.74, 128.22, 128.51, 128.71, 128.76, 128.94 (d, *J*_C−F_ = 31.00 Hz),, 129.01, 129.27, 133.65, 133.76, 135.75, 136.38, 138.38, 144.42, 145.31 (d, *J*_C−F_ = 112.07 Hz), 162.19, 164.16 (d, *J*_C−F_ = 48.85 Hz).

### Optical sensing of ammonia and chromium ions

Optical detection of hexavalent chromium, aqueous ammonia, and other heavy metal ions was evaluated through freshly prepared **compound 4a,b** using the UV–visible spectrophotometer Shimadzu, UV-1800 (Japan). To investigate the prepared **compound 4a,b** interaction with different metals like ZnCl_2_, AlCl_3_, HgCl_2_, NiCl_2_, MnCl_2_, SnCl_2_ about 20 ppm stock solution of these metal ions were prepared. For sensing of chromium, 1.5 mL of different chromium solution concentrations (0–14 ppm) was added to a 4 mL quartz cuvette. About 1.5 mL of freshly prepared **compound 4a,b** (20 ppm) was introduced into a cuvette. The increase in intensity and changes in absorbance peaks at 549 nm of **compound 4a,b** after 3 min of the addition of chromium was monitored by UV–visible spectrophotometer. The same procedure was used for sensing aqueous ammonia. Different concentrations of ammonia were used for sensing procedures, as follows: 0–20 ppm. All experiments were performed in triplicate.

### Characterizations of prepared compounds

Melting points were determined using an Electrothermal (variable heater) melting point apparatus. The NMR spectra were measured with a JEOLNMR 500 MHz spectrometer. ^1^H (500 MHz) and ^13^C NMR (125 MHz) spectra were recorded in deuterated dimethyl sulfoxide (DMSO-*d*_*6*_) using tetramethylsilane as a standard. The chemical shift (δ) was reported in ppm and the chemical shift (*J*) was reported in Hz. The UV–VIS spectrum was recorded using Shimadzu Spectrophotometer.

## Results and discussion

**Scheme 1:**
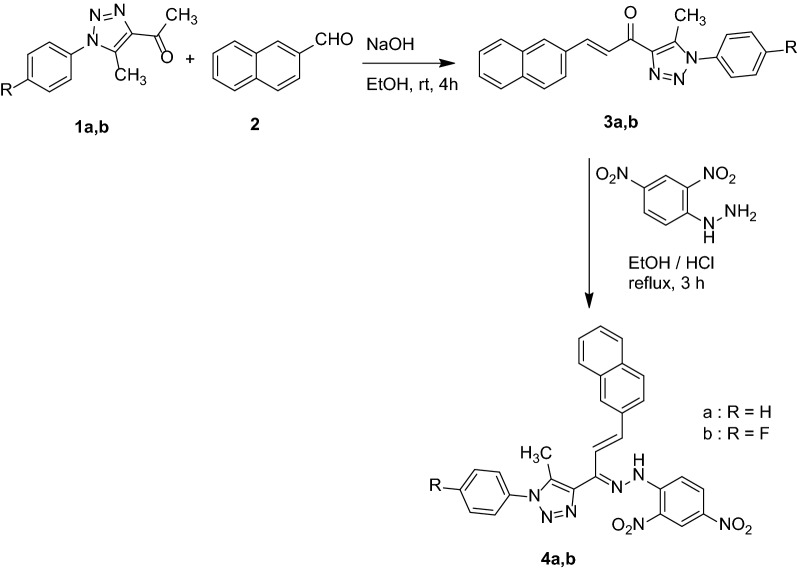
Synthetic routes to allylhydrazones **4a,b**

Aldol condensation reaction of 4-acetyl-1,2,3-triazoles **1a,b** (Ar = C_6_H_4_; 4-FC_6_H_4_) with 2-naphthaldehyde **2** in ethanolic sodium hydroxide at room temperature for 4 h. afforded 1-(5-methyl-1-aryl-1* H*-1,2,3-triazol-4-yl)-3-(naphthalen-2-yl)prop-2-en-1-ones **3a,b** in 93–95% yield. The confirmation of **3a,b** chemical structure was investigated with different spectral techniques like ^1^H NMR and ^13^C NMR (Additional file 1: Figs. S3–S6). The condensation of **3a,b** with (2,4-dinitrophenyl)hydrazine in dry ethanol containing drops from concentrated hydrochloric acid under reflux conditions for 3 h. Furnished novel 4-(1-(2-(2,4-dinitrophenyl)hydrazineylidene)-3-(naphthalen-2-yl)allyl)-5-methyl-1-aryl-1* H*-1,2,3-triazoles (Ar = C_6_H_4_; 4-FC_6_H_4_) **4a,b** in about 88% yield (Scheme 1). The ^1^H NMR and ^13^C NMR for both compounds 4a,b were measured and the spectrum showed all related peaks for confirmation the chemical structure of prepared compounds except peaks at 2.5 and 3.5 ppm are attributed to the DMSO-d_6_ and water, also additional peaks at 2.6, 2.8 and 7.7 ppm are attributed to DMF molecule in the synthesized compounds (Additional file 1: Figs. S7–S10). The peaks at 11.7 and 12.6 are attributed to the product having two isomers which each one showing a different peak, the ^1^H NMR in D_2_O was tested for confirming the exchangeable hydrogens in the compound 4b (Additional file 1: Fig. S11). The functional groups in compounds 4a were tested using FTIR techniques, peaks at 1513, 1604, 2927, 3056, 3406 cm^− 1^ are attributed to C=C, C=N, C–H (aliphatic), C–H (aromatic), and NH, respectively (Additional file 1: Fig. S12). Moreover, the FTIR spectrum for compounds 4b showed peaks for characteristic groups NH, C–H (aromatic), C–H (aliphatic), C=N, and C=C at 3315, 3026, 2935, 1612, and 1515 cm^− 1^, respectively (Additional file 1: Fig. S13).

The chemical structures of **3a,b**, and **4a,b** were established by the NMR spectral data. The ^1^H NMR spectrum of **3b** showed a characteristic two doublet singles at δ 7.28 and 7.51 ppm with a coupling constant 8.6 Hz corresponding to the olefin protons. While the (NH) proton in the compound of **4b** appeared as a singlet divided into two peaks at 11.70 and 12.56 ppm due to the effect of the double bond. The ^13^C NMR spectrum of compound **3a** showed a carbonyl group at 184.36 ppm.

### Sensing study and selectivity

**Fig. 1:**
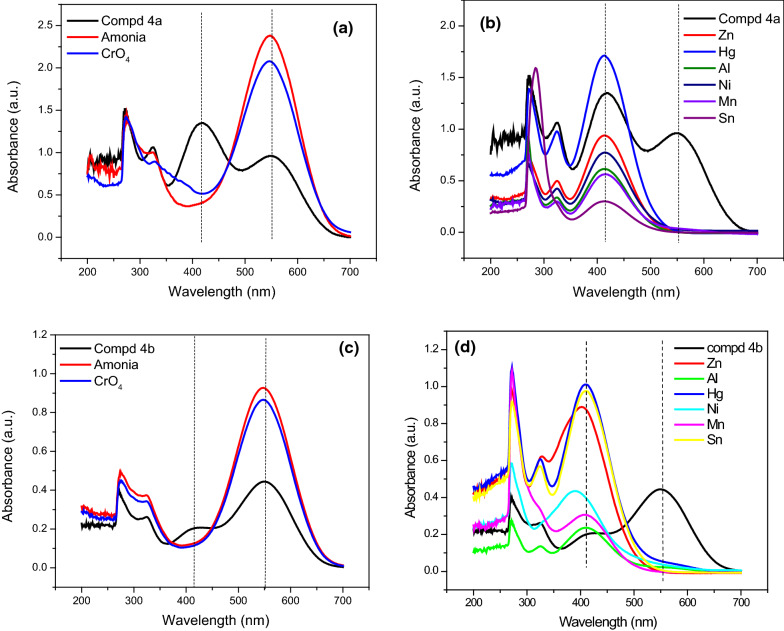
UV–Vis spectra of **compound 4a** in the presence of **a** NH_4_OH and K_2_CrO_4_ and **b** ZnCl_2_, AlCl_3_, HgCl_2_, NiCl_2_, MnCl_2_, SnCl_2_ and **compound 4b** in the presence of **c** NH_4_OH and K_2_CrO_4_ and **d** ZnCl_2_, AlCl_3_, HgCl_2_, NiCl_2_, MnCl_2_, SnCl_2_

After adding varying concentrations of ammonia and other harmful heavy metal ions to compound 4a,b solutions, the color changes were observed using a UV–visible spectrophotometer. After 3 min, the UV–visible spectra of each species were recorded. Figure 1 depicts the color shift of 4a,b solutions. When ammonia and chromium ions were tested, the dark violet color of compounds 4a,b changed dramatically (Fig. 1a, c). The spectrum of compounds 4a,b was identical, with two prominent distinctive peaks at 418 and 549 nm. The catalytic dissociation of compounds 4a,b is responsible for the selective detection of chromium ions and ammonia. Due to the hydrolysis behavior of both chromium and ammonia, the peak at 418 nm vanished and the peak at 549 nm was predominated. The aqueous solutions containing different heavy metal ions ZnCl_2_, AlCl_3_, HgCl_2_, NiCl_2_, MnCl_2_, and SnCl_2_ interacted with the synthesized compound 4a,b (Fig. 1b, d). Following 3 min of interaction with compounds 4a,b, the UV–visible spectra of compounds 4a,b were collected after the addition of metal ions. The UV–visible spectra of compounds 4a,b changed, with the peak at 549 nm disappearing and a peak at 418 nm predominated.

### Sensing of ammonia

**Fig. 2:**
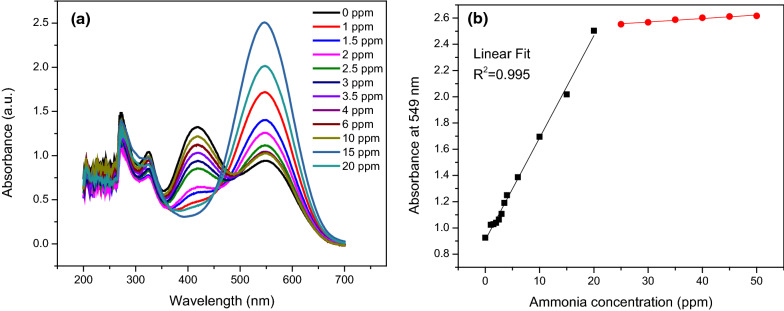
**a** The plot of changes in UV–visible spectra of **compound 4a** solution as a function of different ammonia concentrations (0–20 ppm), **b** the linear relationship between different ammonia concentrations and absorbance intensity at 549 nm wavelength

The optical measurement with a UV–visible spectrophotometer was used to detect ammonia solution. As shown in Fig. 2, the variations in the spectra of produced compound 4a were studied with various doses of ammonia (0–20 ppm). The changes in the absorbance value of compound 4a as a function of ammonia concentration were recorded. The intensity of compound 4a increases when the ammonia concentration rises from 0 to 20 ppm, as seen in Fig. 2. Because varying ammonia concentrations altered the peak at 549 nm, the absorption spectra at 549 nm may be easily studied and the ammonia content identified. The absorption peak at 418 nm in compound 4a could be owing to the chemical’s connection with ammonia. As shown in Fig. 2b, the kinetics of the reaction were determined by processing the data and plotting the absorbance amount at 549 nm wavelength vs. ammonia concentration. With a correlation factor R^2^ of 0.995, the relative change in absorbance provides an excellent linear correlation from 0 to 20 ppm. In the range from 20 to 50 ppm, the diagram gives a horizontal line due to the saturation of the solution. The saturation point shows the stoichiometry of compounds against ammonia. Additional file 1: Fig. S1 shows the change of ammonia uptake for compound 4b at different concentrations. Compound 4b has similar behavior to compound 4a, but compound 4a is more sensitive to ammonia.

### Sensor of chromium

**Fig. 3:**
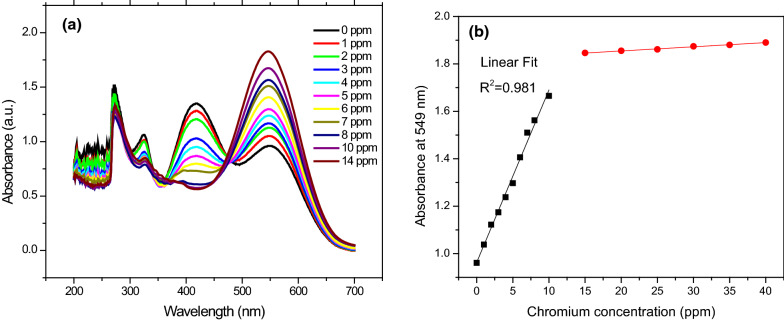
**a **Plot of change in the UV–visible spectra of **compound 4a** at different concentrations of chromium ions; **b** the linear relationship between chromium concentration and absorbance intensity

Using a UV–visible spectrophotometer, the probable mechanism of interaction of produced compound 4a,b with chromium ions was investigated. After adding potassium chromate, the color of the compound 4a,b solutions changed dramatically to dark blue, as illustrated in Fig. 3. For the chromium ions sensing using compounds 4a,b, different amounts of chromate anion (0–14 ppm) were utilized. Compound 4a,b (2 mL, 20 ppm) was placed in a 4 mL quartz cuvette, followed by 1 mL of potassium chromate solution, and their UV–visible spectra were monitored using a UV–visible spectrophotometer. The peak at 418 nm disappeared after the chromate solution was added to the cuvette containing compound 4a,b, and the peak at 549 nm developed, with the strength increasing as the concentration of chromium increased. The linear association between chromium content and compound 4a absorbance intensity was shown in Fig. 3b. However, the linear relationship between compound 4b and the concentration of chromium ions were present in Additional file 1: Fig. S2.

### Mechanism of ammonia and heavy metals sensing

**Scheme 2:**
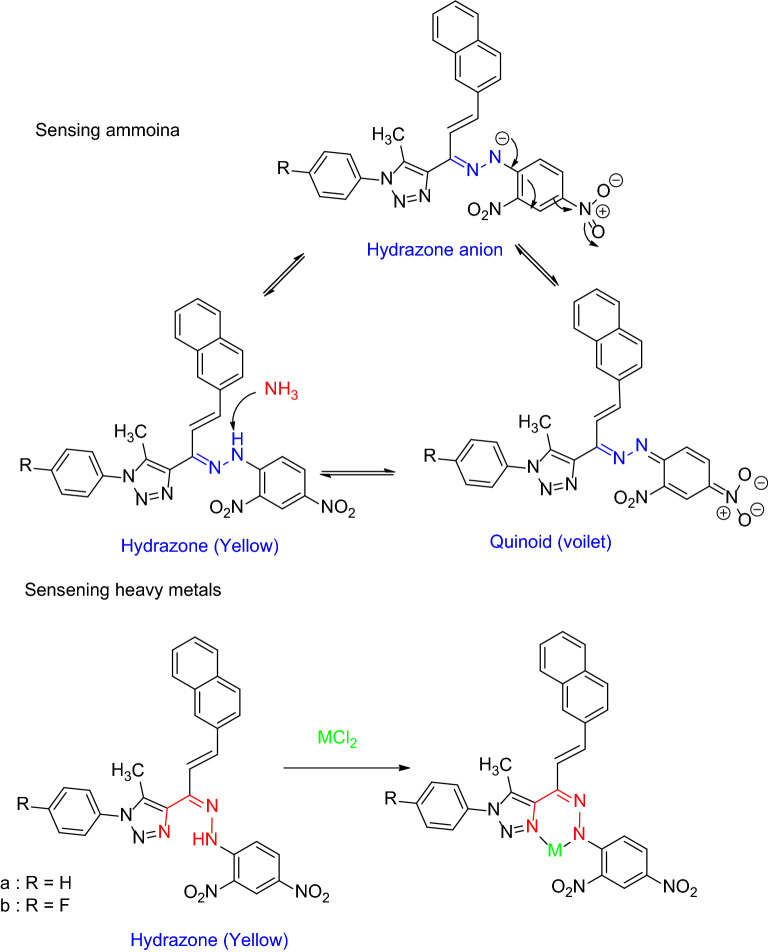
The proposed mechanism for sensing ammonia and heavy metals

The ammonia sensing mechanism is related to convert of hydrazone to quinoid [26]. Compound 4a,b has 2,4-dinitrophenylhydrazone in its backbone molecular structure which makes the existing switch with ammonia molecule (Scheme 2). Ally hydrazone represented an attractive molecule for heavy metal chemosensors and the main mechanism was coordination behavior [27–30]. Here in our work, the quenching effect was measured for sensing heavy metals. The metal ions were coordinated with a free nitrogen atom center with free lone pair of the electron as shown in Scheme 2.

**Fig. 4:**
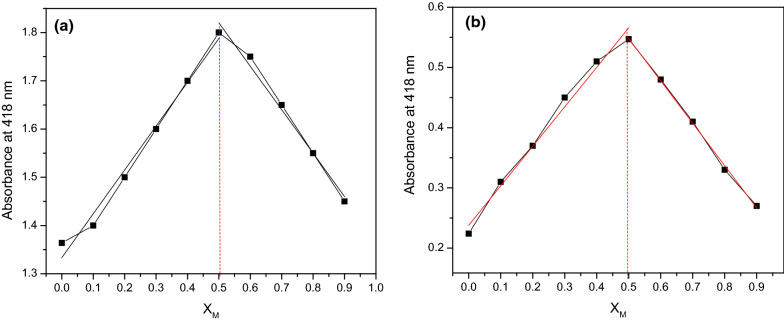
Job’s plot for the determination of the binding stoichiometry of **a** compound 4a with Hg^2+^ obtained from variations in absorption at 418 nm, **b** compound 4b with Hg^2+^ obtained from variations in absorption at 418 nm

The chelation of heavy metals for compounds 4a,b were studied by continuous variance (Job’s plot analysis). This was done by carefully changing the molecular fractions of compounds 4a,b against the Hg^2+^ ions (the interesting metal due to high absorption density). To understand the binding behavior of compounds 4a,b with mercury ions and determination of the stoichiometry for the formed complex, the absorption of the resulting complex was studied using UV–visible spectroscopy. The absorption intensity at 418 nm increases at the initial stage, then at 0.5 decreases as the mole fraction of Hg^2+^ ions increases. The Job’s plot for the adsorption was determined by keeping the sum of the initial concentrations of Hg^2+^ and compounds 4a,b constant at 10 µM and changing the molar ratio of Hg^2+^ (X_M_ = ([Hg^2+^]/([Hg^2+^] + [compounds 4a,b])) from 0 to 1. Job’s plot for the molecular fraction was plotted for Hg^2+^ ions (Fig. 4a, b) giving about 0.5 (Fig. 4), indicating that the complex formed between compound 4a, b, and Hg^2+^ ions follow a 1:1 stoichiometric measure.

## Conclusions

Hydrazone derivatives are a great chemical compound that may be utilized to detect a variety of ions, including ammonia and heavy metal ions. As a result, compound 4a,b was synthesized using a green approach and used as a hydrazone scaffold in the development of optical sensors. Different spectrum techniques, such as NMR, FTIR, and UV–VIS, were used to test compounds 4a,b. The optical determination of ammonia and chromium ions was performed using the synthesized compound 4a,b. In the range of 0–20 ppm, the synthesized compounds displayed high selectivity for ammonia and chromium ion detection.

## Supplementary Information

**Additional file 1: Figure S1.** [a] Change of ammonia absorbance for compound 4b with different concentration; [b] linear fitting. **Figure S2.** [a] Change of chromium absorbance for compound 4b with different concentration; [b] linear fitting. **Figure S3.** NMR spectrum (^13^C NMR) for compound 3a. **Figure S4.** NMR spectrum (^1^H NMR) for compound 3a. **Figure S5.** NMR spectrum (^13^C NMR) for compound 3b. **Figure S6.** NMR spectrum (^1^H NMR) for compound 3b. **Figure S7.** NMR spectrum (^13^C NMR) for compound 4a. **Figure S8.** NMR spectrum (^1^H NMR) for compound 4a. **Figure S9.** NMR spectrum (^13^C NMR) for compound 4b. **Figure S10.** NMR spectrum (^1^H NMR) for compound 4b. **Figure S11.** NMR spectrum (^1^H NMR) for compound 4b in D_2_O. **Figure S12.** FTIR spectrum for compound 4a. **Figure S12.** FTIR spectrum for compound 4b.

## Data Availability

The data are available in a additional file.
