# Supplementary material for: Novel allyl-hydrazones including 2,4-dinitrophenyl and 1,2,3-triazole moieties as optical sensor for ammonia and chromium ions in water
Source: BMC Chem. 2022 Apr 7;16(1):26. doi: 10.1186/s13065-022-00820-2 (PMC8991505; doi:10.1186/s13065-022-00820-2)
Supplement: Supplementary file 1 — Additional file 1: Figure S1. [a] Change of ammonia absorbance for compound 4b with different concentration; [b] linear fitting. Figure S2. [a] Change of chromium absorbance for compound 4b with different concentration; [b] linear fitting. Figure S3. NMR spectrum (13C NMR) for compound 3a. Figure S4. NMR spectrum (1H NMR) for compound 3a. Figure S5. NMR spectrum (13C NMR) for compound 3b. Figure S6. NMR spectrum (1H NMR) for compound 3b. Figure S7. NMR spectrum (13C NMR) for compound 4a. Figure S8. NMR spectrum (1H NMR) for compound 4a. Figure S9. NMR spectrum (13C NMR) for compound 4b. Figure S10. NMR spectrum (1H NMR) for compound 4b. Figure S11. NMR spectrum (1H NMR) for compound 4b in D2O. Figure S12. FTIR spectrum for compound 4a. Figure S12. FTIR spectrum for compound 4b. [file 13065_2022_820_MOESM1_ESM.docx]

Additional file

Figure S1. [a] Change of ammonia absorbance for compound 4b with different concentration; [b] linear fitting

Figure S2.[a] Change of chromium absorbance for compound 4b with different concentration; [b] linear fitting


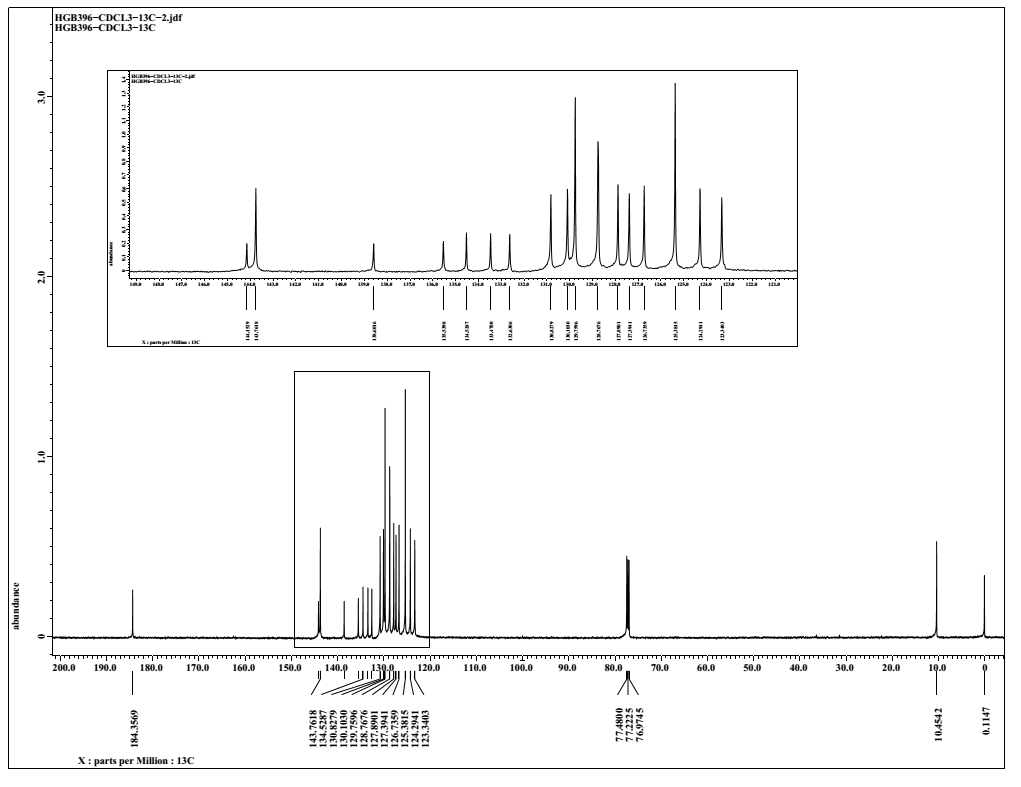


Figure S3. NMR spectrum (^13^ C NMR) for compound 3a


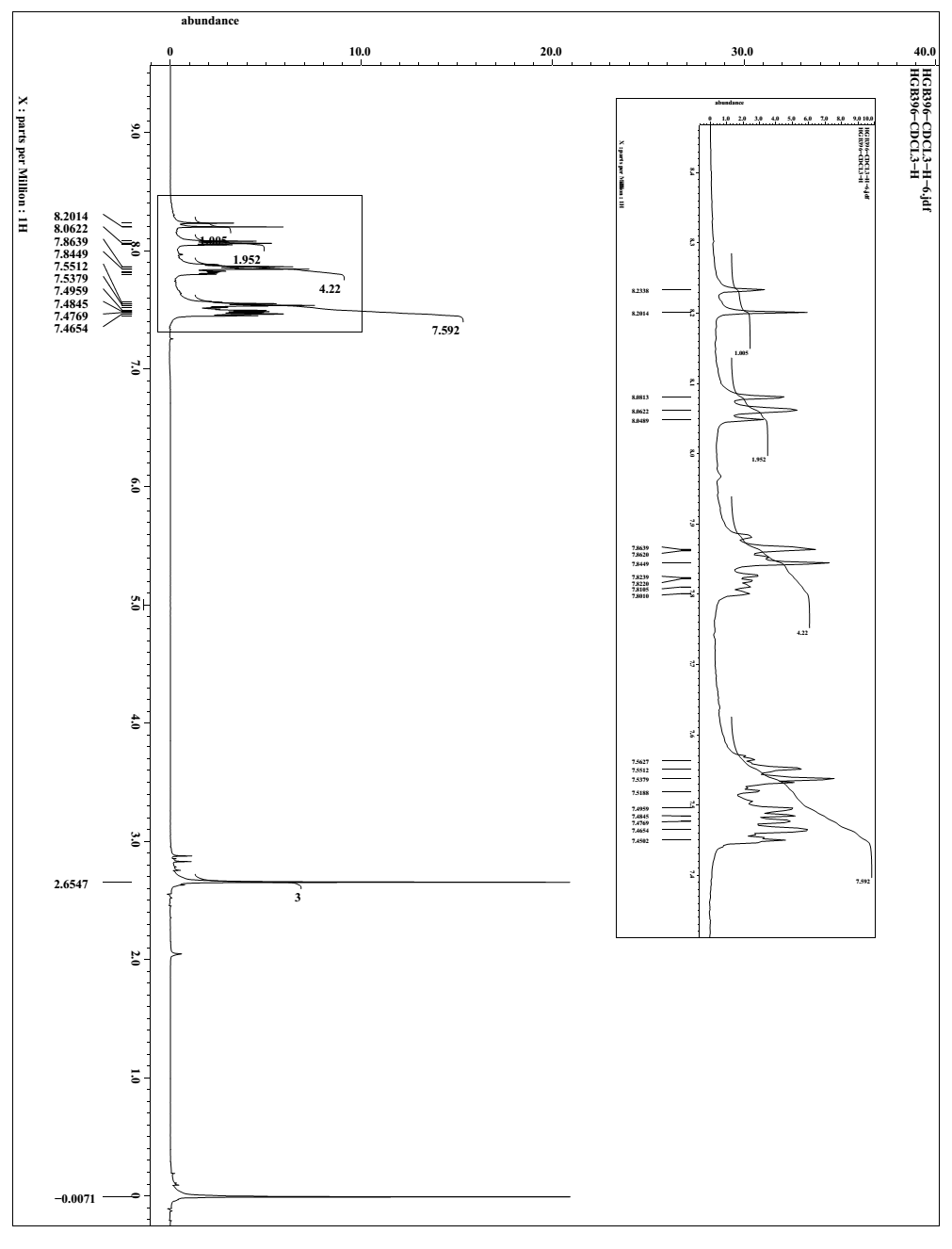


Figure S4. NMR spectrum (^1^ H NMR) for compound 3a


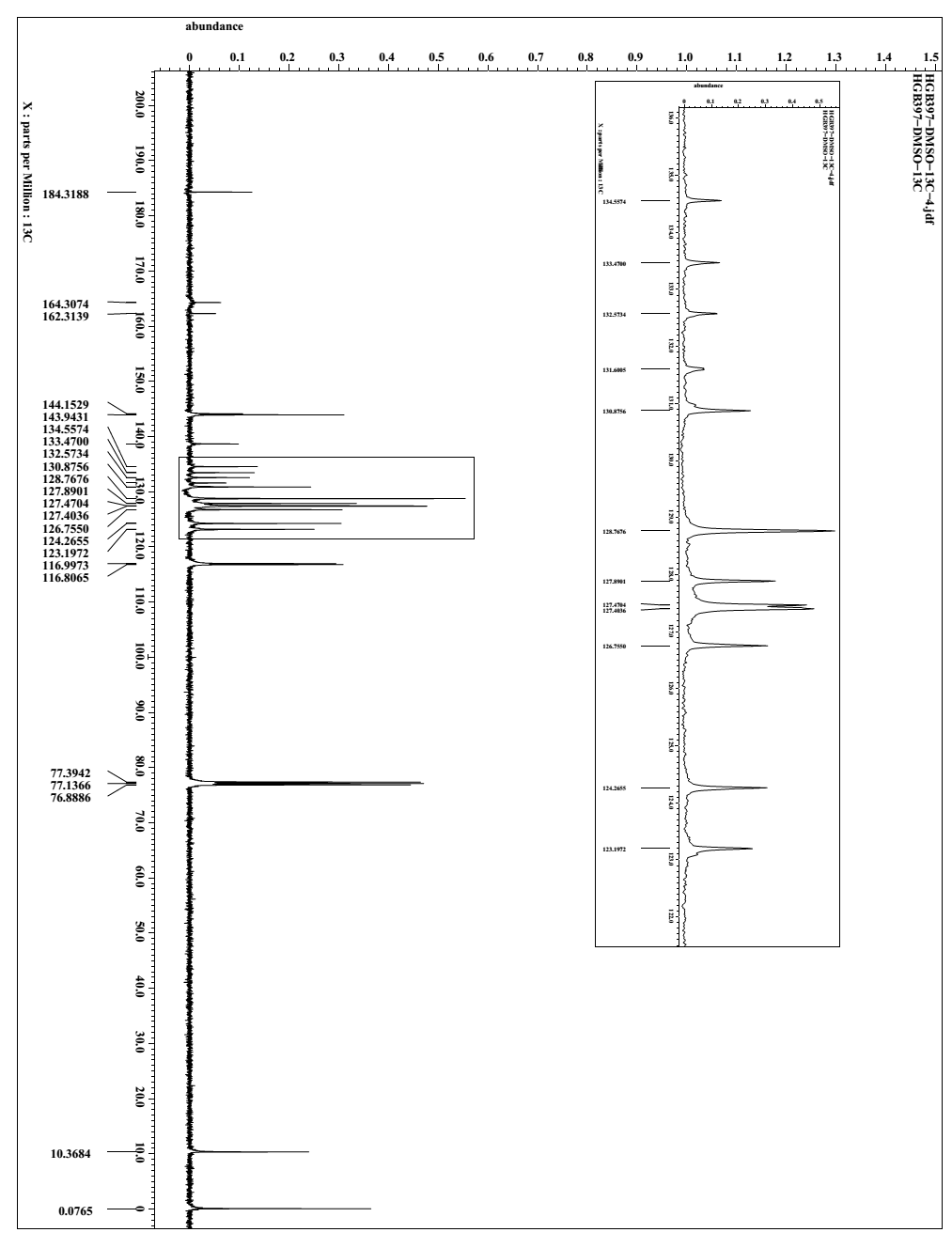


Figure S5. NMR spectrum (^13^ C NMR) for compound 3b


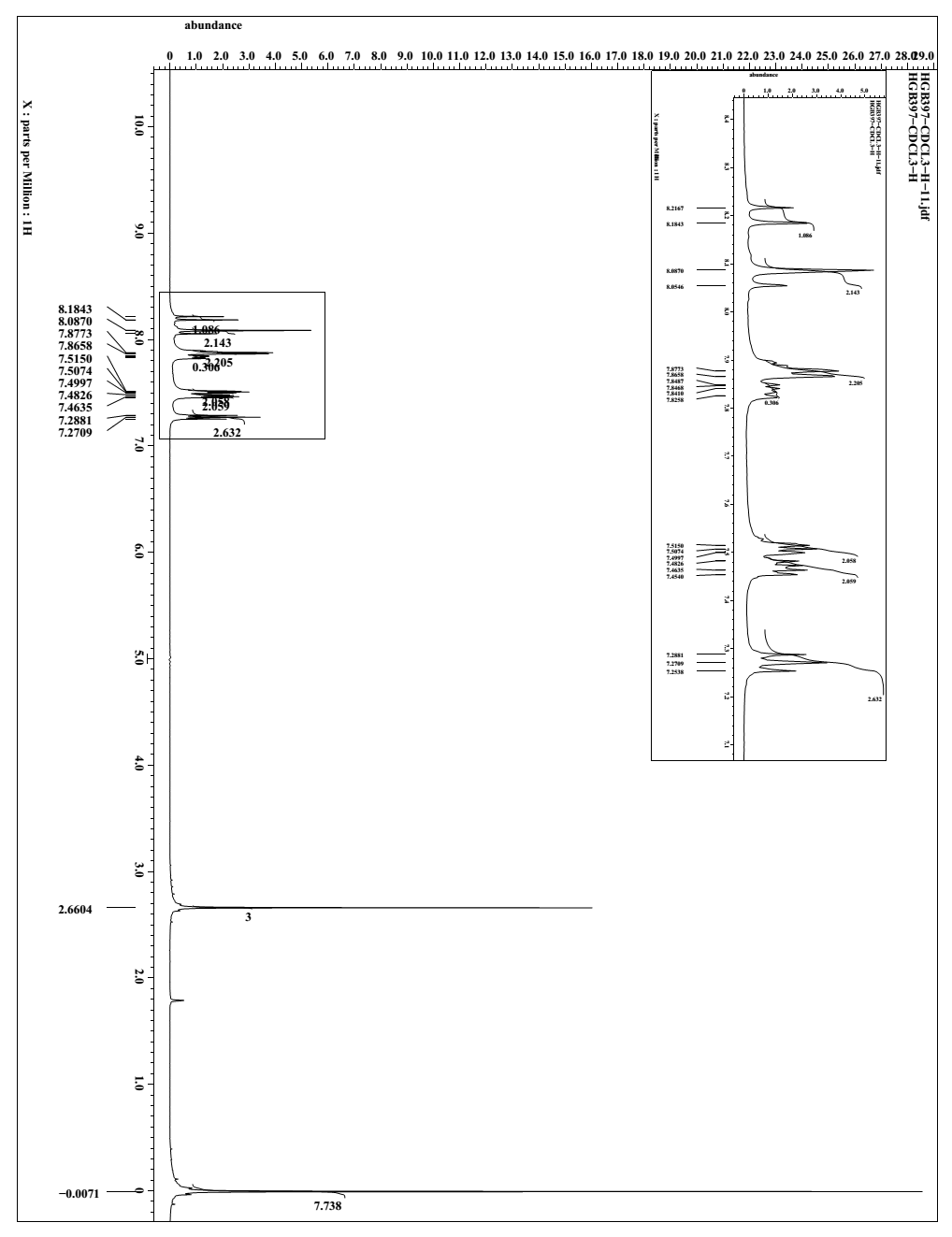


Figure S6. NMR spectrum (^1^ H NMR) for compound 3b


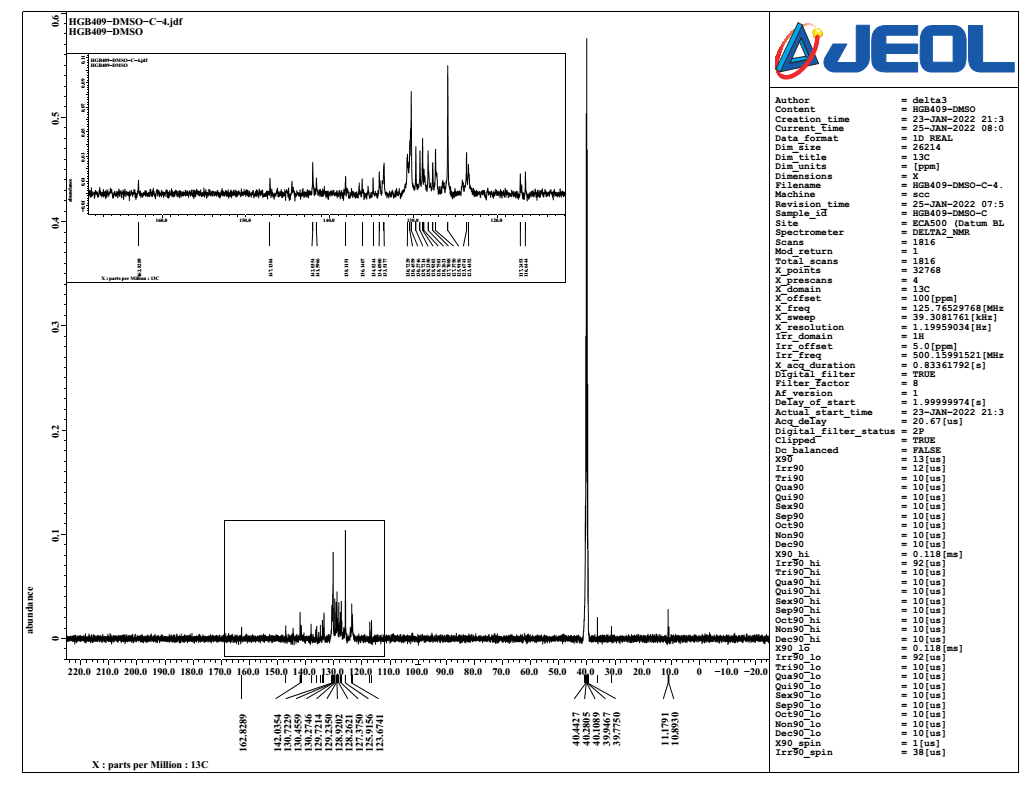


Figure S7. NMR spectrum (^13^ C NMR) for compound 4a


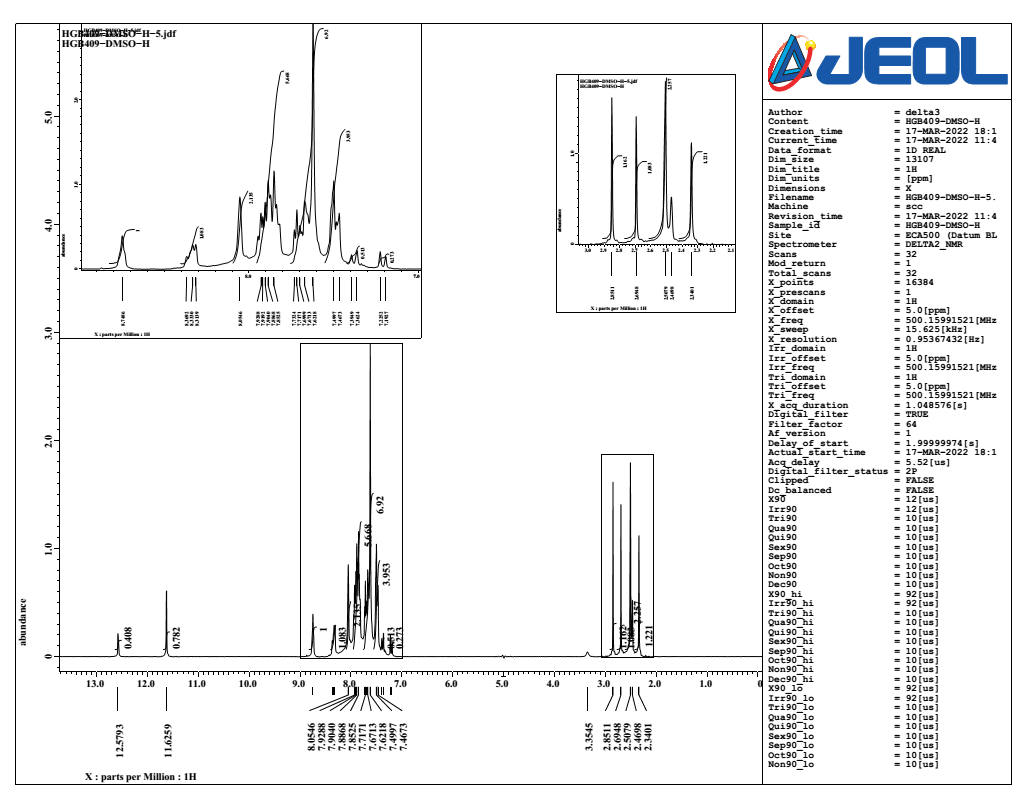


Figure S8. NMR spectrum (^1^ H NMR) for compound 4a


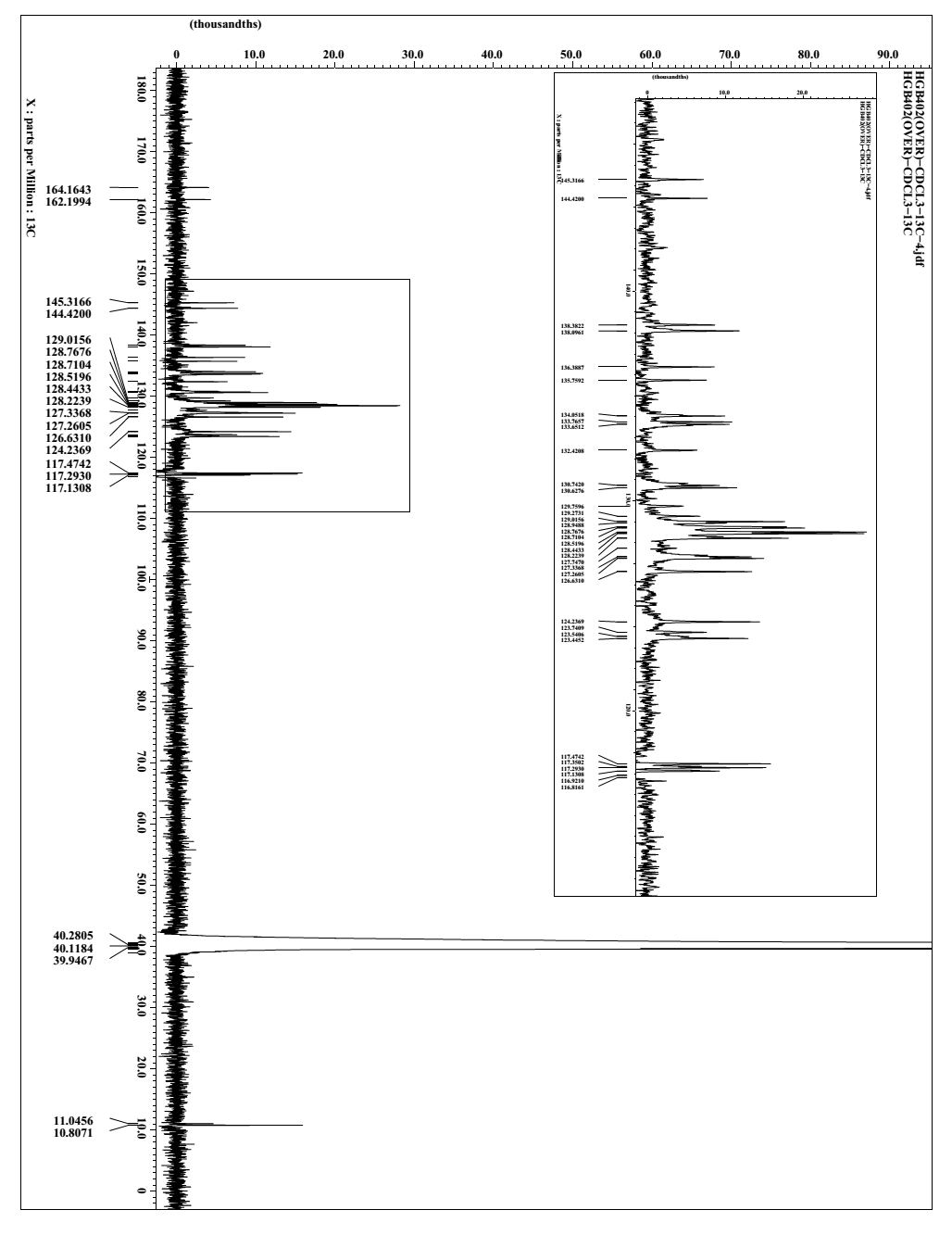


Figure S9. NMR spectrum (^13^ C NMR) for compound 4b


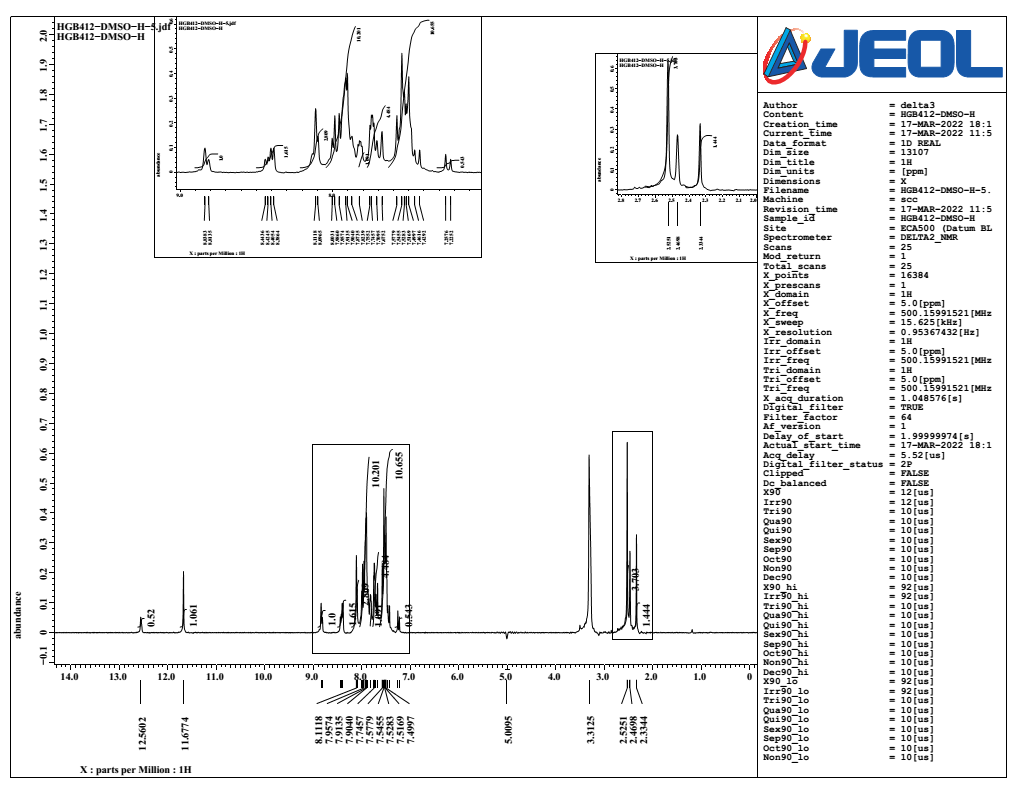


Figure S10. NMR spectrum (^1^ H NMR) for compound 4b


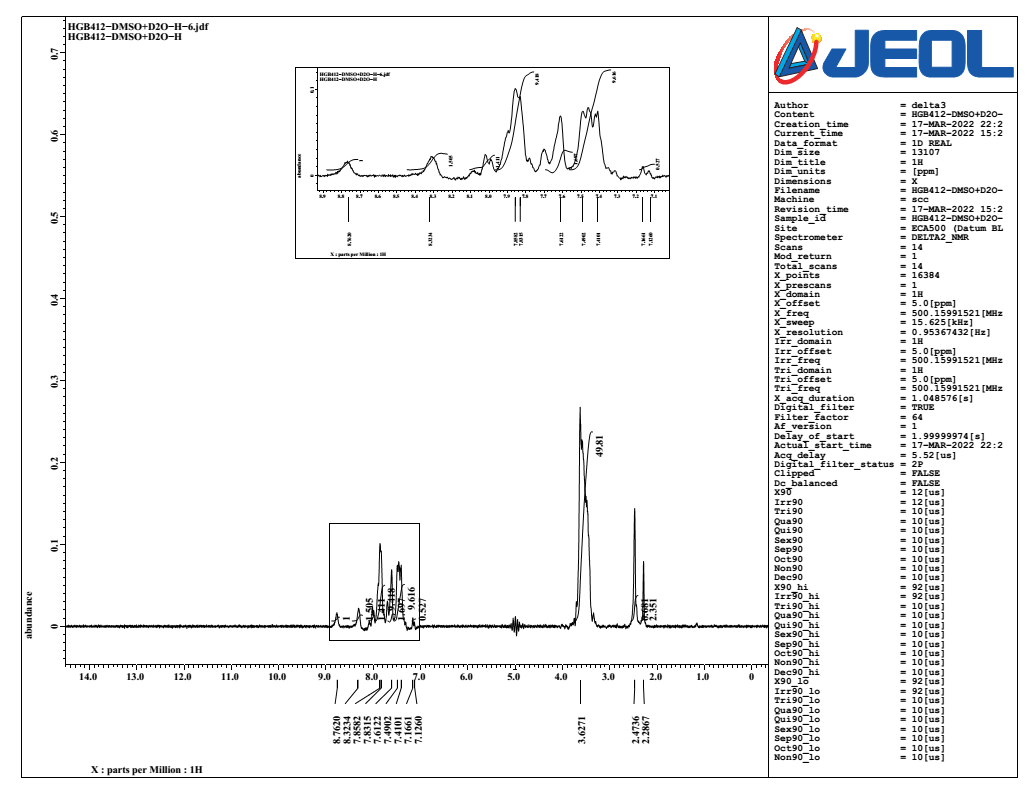


Figure S11. NMR spectrum (^1^ H NMR) for compound 4b in D_2_O

Figure S12. FTIR spectrum for compound 4a

Figure S12. FTIR spectrum for compound 4b
